# Naturally Acquired Humoral Immunity against Malaria Parasites in Non-Human Primates from the Brazilian Amazon, Cerrado and Atlantic Forest

**DOI:** 10.3390/pathogens9070525

**Published:** 2020-06-29

**Authors:** Eliana Ferreira Monteiro, Carmen Fernandez-Becerra, Maisa da Silva Araujo, Mariluce Rezende Messias, Luiz Shozo Ozaki, Ana Maria Ribeiro de Castro Duarte, Marina Galvão Bueno, Jose Luiz Catao-Dias, Carolina Romeiro Fernandes Chagas, Bruno da Silva Mathias, Mayra Gomes dos Santos, Stéfanie Vanessa Santos, Marcia Moreira Holcman, Julio Cesar de Souza, Karin Kirchgatter

**Affiliations:** 1 Instituto de Medicina Tropical, Faculdade de Medicina, Universidade de São Paulo, São Paulo, SP 05403-000, Brazil; elianafmonteiro@usp.br (E.F.M.); amrcd2@gmail.com (A.M.R.d.C.D.); brunomathiasbio@gmail.com (B.d.S.M.); 2 ISGlobal, Hospital Clínic—Universitat de Barcelona, 08036 Barcelona, Spain; carmen.fernandez@isglobal.org; 3 Germans Trias i Pujol Health Science Research Institute (IGTP), 08916 Badalona, Spain; 4 Instituto Oswaldo Cruz, Fundação Oswaldo Cruz, Fiocruz Rondônia, Porto Velho, RO 76812-245, Brazil; maisaraujo@gmail.com; 5 Departamento de Biologia, Universidade Federal de Rondônia, Porto Velho, RO 78900-000, Brazil; messias.malu@gmail.com; 6 Life Sciences, Virginia Commonwealth University, Richmond, VA 23284, USA; lsozaki@vcu.edu; 7 Departamento de Laboratórios Especializados, Superintendência de Controle de Endemias, São Paulo, SP 01027-000, Brazil; marciaholcman@gmail.com; 8 Instituto Oswaldo Cruz, Fundação Oswaldo Cruz, Fiocruz Rio de Janeiro, Rio de Janeiro, RJ 21040-900, Brazil; buenomg@gmail.com; 9 Departamento de Patologia, Faculdade de Medicina Veterinária e Zootecnia, Universidade de São Paulo, São Paulo, SP 05508-270, Brazil; zecatao@usp.br; 10 Departamento de Pesquisas Aplicadas, Fundação Parque Zoológico de São Paulo, São Paulo, SP 04301-905, Brazil; crfchagas@gmail.com; 11 Institute of Ecology, Nature Research Centre, Vilnius 08412, Lithuania; 12 Departamento de Patologia, Universidade Cruzeiro do Sul, São Paulo, SP 01311-925, Brazil; mayra.santos_01@hotmail.com (M.G.d.S.); stefanie@stefanie.vet.br (S.V.S.); 13 Departamento de Anatomia Patológica, AC Camargo Cancer Center, São Paulo, SP 01525-001, Brazil; 14 Departamento de Medicina Veterinária, Fundação Universidade Regional de Blumenau, Blumenau, SC 89012-900, Brazil; juliosouzavet@gmail.com; 15 Projeto Bugio, Centro de Pesquisas Biológicas, Indaial, SC 89130-000, Brazil

**Keywords:** malaria, *Plasmodium malariae*, MSP1, non-human primates, serology, Brazil

## Abstract

Non-human primates (NHPs) have been shown to be infected by parasites of the genus *Plasmodium*, the etiological agent of malaria in humans, creating potential risks of zoonotic transmission. *Plasmodium brasilianum*, a parasite species similar to *P. malariae* of humans, have been described in NHPs from Central and South America, including Brazil. The merozoite surface protein 1 (MSP1), besides being a malaria vaccine candidate, is highly immunogenic. Due to such properties, we tested this protein for the diagnosis of parasite infection. We used recombinant proteins of *P. malariae* MSP1, as well as of *P. falciparum* and *P. vivax*, for the detection of antibodies anti-MSP1 of these parasite species, in the sera of NHPs collected in different regions of Brazil. About 40% of the NHP sera were confirmed as reactive to the proteins of one or more parasite species. A relatively higher number of reactive sera was found in animals from the Atlantic Forest than those from the Amazon region, possibly reflecting the former more intense parasite circulation among NHPs due to their proximity to humans at a higher populational density. The presence of *Plasmodium* positive NHPs in the surveyed areas, being therefore potential parasite reservoirs, needs to be considered in any malaria surveillance program.

## 1. Introduction

The merozoite surface protein 1 (MSP1) is the most abundant protein on the malaria parasite cell surface. It is involved in the erythrocyte invasion process, being one of the most studied targets for a malaria vaccine [1]. MSP1 is synthesized from the onset of schizogony [2] and transported to the surface of the parasite cell, together with the proteins MSP6 and MSP7 as a complex, where it is retained through its glycosyl phosphatidylinositol (GPI) anchor. The egress of merozoites from erythrocytes is accompanied by a primary proteolytic processing of MSP1 that results in a protein complex on the surface of the merozoite cells (reviewed in [1]).

The gene of *Plasmodium malariae* MSP1 (*pmmsp1*) encodes a protein of 1751 amino acids, including a 19 amino acid signal peptide [3]. Recently, we analyzed a portion of this gene in *P. malariae*, and in its non-human primate (NHP) equivalent, *P. brasilianum*, isolated from different geographic regions of Brazil [4]. Analyses of the *pmmsp1* gene was performed through the amplification and sequencing of five fragments, F1 to F5 (F5 also named PmMSP1_19_), equivalent to blocks 3 to 17 of the PfMSP1 (*P. falciparum* MSP1) and covering 60% of the gene [4]. Except for the fragment F4, all others showed differences to a previously published sequence [4], the PmMSP1 allele MM1A of a parasite isolated from a patient in Cameroon [3]. The most polymorphic region was in the sequence of fragment F2, previously described to include imperfect repeats [3].

The availability of recombinant proteins from *P. falciparum* and *P. vivax* has allowed for numerous studies revealing whether individuals have been naturally exposed to malaria parasites, by detecting antibodies to the protein in their sera (reviewed in [5]). For the purposes of studying such exposure in NHPs, we produced recombinant glutathione S-transferase (GST)-fusion proteins out of the characterized *P. malariae msp1* gene fragments, named PmMSP1_F1_, PmMSP1_F2_, PmMSP1_F3_, PmMSP1_F4_ and PmMSP1_19_. The immunization of BALB/c mice with these recombinant proteins elicited a significant humoral immune response, making them potential component candidates for a vaccine against *P. malariae.* These recombinant proteins were also shown to be very useful as diagnostic markers in epidemiological studies and for the differential diagnosis of *P. malariae* infection [6].

In this work, we evaluated the diagnostic capability of the *P. malariae* MSP1 recombinant proteins, together with the MSP1_19_ of *P. falciparum* (PfMSP1_19_) and of *P. vivax* (PvMSP1_19_), for the detection of anti-MSP1 in the sera of NHPs from malaria endemic regions of Brazil, the Amazon and Atlantic forests, and from a non-endemic region, the Cerrado, in Central Brazil. Using these recombinant proteins, we also aimed to find the prevalence of antibodies against these parasite species in the sera of NHPs and, thus, the potentiality of these animals as malaria reservoirs.

## 2. Materials and Methods

### 2.1. Sera Sampling from Non-Human Primates

**Figure 1:**
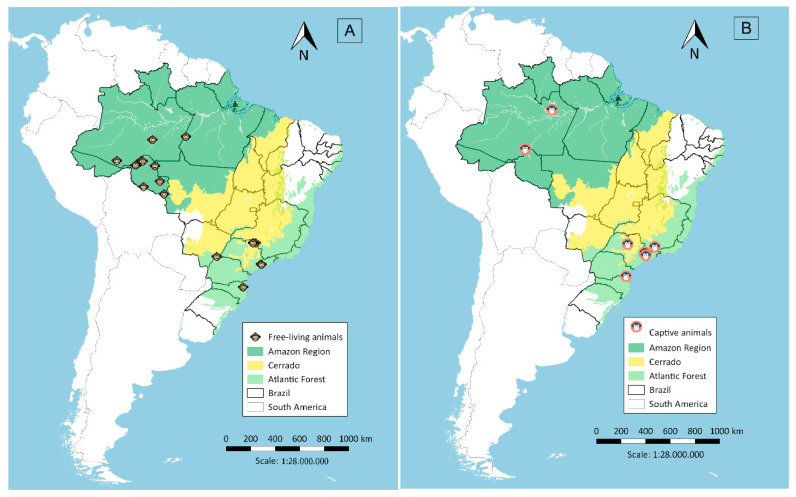
Sera collection sites in the Amazon Region, Cerrado and the Atlantic Forest, Brazil, from free-living (**A**) and captive animals (**B**).

Serum samples were collected in two malaria endemic regions in Brazil, the Amazon and Atlantic Forest regions, and in a non-endemic region, the Cerrado region in Central Brazil. A total of 373 samples were collected from free-living animals and 122 from captive animals. Sera from free-living animals (Figure 1A) were obtained in different localities: (i) in the Amazon Region (*n* = 155), collected close to Porto Velho city in Rondônia state, from March 2009 to November 2012, during projects related to environmental management for the construction of hydroelectric power plants [7,8]; (ii) in the Atlantic Forest (*n* = 111), from October 1997 to July 2005, in forest fragments around the municipality of São Paulo [9] and the municipality of Indaial in Santa Catarina state, from June 2001 to February 2015; and (iii) in the Cerrado region (*n* = 107), from April 2000 to March 2001 and January to December 2009, in the canopy of woods in flooded areas of the lake at Porto Primavera dam during wildlife rescue operations [10]. Sera from captive animals (Figure 1B) were from the Atlantic Forest area (*n* = 103), 60 being from the São Paulo city Zoo, 31 from the Tietê Ecological Park and 12 from CETAS (Centro de Triagem de Animais Silvestres; Wildlife Rescue and Rehabilitation Center) in Lorena city (*n* = 08) and Unimonte in São Vicente city (*n* = 1), as well as from Bauru city Zoo (*n* = 1) and the Centre for Biological Research in Indaial city (*n* = 2). From the Amazon Region (*n* = 19), samples were collected from animals kept close to areas of human habitation, such as the Ecological Park of Porto Velho city, and animals rescued by IBAMA (Brazilian Institute of Environment and Renewable Natural Resources) which had been kept illegally as pets in rural or suburban areas.

All procedures were approved by the Ethical Committee in Animal Research (CEUA) of the Institute of Tropical Medicine of São Paulo, University of Sao Paulo (number 2014/281A) and were in full compliance with federal permits issued by the Brazilian Ministry of the Environment (SISBIO numbers 14081, 17302, 18861, 24319, 44751, 47812, 50076).

### 2.2. Recombinant Antigens

GST and GST-recombinant fusion proteins of PmMSP1 (PmMSP1_F1_, PmMSP1_F2_, PmMSP1_F3_, PmMSP1_F4_, PmMSP1_19_), *P. vivax* (PvMSP1_19_) and *P. falciparum* (PfMSP1_19_), representing the polymorphic N-terminal (MSP1_F1_), the central (MSP1_F2_, MSP1_F3_, MSP1_F4_) and the conserved C-terminal (MSP1_19_) regions [6], were used for detecting anti-parasite antibodies in the sera samples. The GST and GST-fusion proteins were purified on glutathione-Sepharose 4B (GE Healthcare, MilliporeSigma, St. Louis, MO, USA) and the protein concentration was determined with the Bradford protein Assay (Bio-Rad, Hercules, CA, USA). 

### 2.3. Multiplexed Serological Assay 

Recombinant proteins were covalently bound to Bio-Plex Pro Magnetic COOH Beads using the BioPlex Amine Coupling Kit (BioPlex Amine Coupling Kit, Bio-Rad, Hercules, CA, USA), following the manufacturer’s instructions. Coupled beads were then used for the analyses of the NHP sera as described [11], with modifications. Briefly, 50 μL of bead suspension, corresponding to 2000 coated beads, was used with each serum sample. Serum samples were diluted 1:50 in assay buffer (PBS 1×, BSA 1%, Tween 20 0.02%) and 50 μL aliquots added to 50 μL of protein coated magnetic beads (final dilution 1:100). Aliquots of 50 μL of biotinylated monkey IgG antibody (MilliporeSigma, St. Louis, MO, USA) (diluted 1:2000) and of phycoerythrin conjugated streptavidin (2 μg/mL) (MilliporeSigma, St. Louis, MO, USA) were used in subsequent incubations. Beads were re-suspended in 125 μL of assay buffer (PBS 1×, BSA 1%, Tween 20 0.02%) and fluorescence measured with the BioPlex200 system (Bio-Rad, Hercules, CA, USA). Results were expressed as median fluorescence intensity (MFI).

Sera were tested in two replicates and positivity evaluated by the measured MFI values of antibodies binding to the recombinant proteins, minus the MFI value of the same serum to non-fusion GST. The cut-off values are presented as the geometric mean of values obtained with a panel of eight negative control sera plus 3× standard deviations. Average cut-off values are shown in Table 1.

### 2.4. Assessment of Coupling Efficiency

To determine the overall efficiency of the *Plasmodium* spp. GST-MSP1 fusion proteins coupling to the BioPlex carboxylated beads, multiplex assays were performed using a rabbit anti-GST polyclonal IgG antibody (Biotin) (Abcam, Cambridge, MA, USA) to detect the fusion protein on the bead. The dilution of the anti-GST antibody was 1:1000 in assay buffer (PBS 1×, BSA 1%, Tween 20 0.02%) (50 μL/well). The bound anti-GST antibody was detected with R-phycoerythrin-labelled streptavidin and fluorescence was measured on the BioPlex 200 instrument (Bio-Rad, Hercules, CA, USA), as described above.

## 3. Results

### 3.1. Testing of Coupling Efficiency of GST-Fusion Proteins to BioPlex Carboxylated Magnetic Beads 

Quality control of the coupling was placed on each plate, where the beads that were covalently coated with the different recombinant proteins were tested in the same plate, with biotinylated anti-GST, to evaluate the efficiency of the coupling and verify the reading behavior of each protein. In Figure S1 (see Supplementary information), it was observed that the reading remained stable and very similar for all the proteins with minimal variation between plates, except for fragment 4 (PmMSP1_F4_), which was unstable and lower in all readings. PvMSP1_19_ presented a similar fluorescence curve but slightly lower, except for Plate 1.

### 3.2. Antibodies Against MSP1 in the NHP Sera

A total of 495 NHP serum samples were analyzed, belonging to the two groups: (i) free living animals (*n* = 373); (ii) captive animals (*n* = 122). Of all samples analyzed, 199 (40.2%) presented IgG antibodies against at least one of the malaria recombinant proteins. Sera that were reactive to the *P. malariae* PmMSP1_F1_ were most frequent, with a total of 109 (22%), followed by those reactive to *P. vivax* MSP1_19_ (19.8%). The least reactive sera were to *P. falciparum* PfMSP1_19_ (1.6%) (Table 2, Tables S1 and S2). 

**Figure 2:**
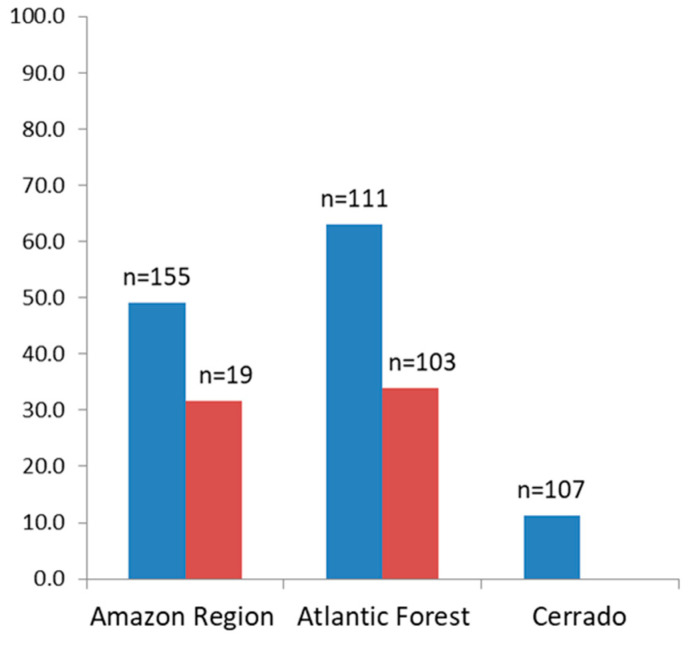
Percentage of positive sera from free-living (blue) and from captive (red) non-human primates to one or more of the MSP1 recombinant proteins.

The percentage of sera of free-living animals reacting to the recombinant proteins was higher in animals from the Atlantic Forest (63.1%), compared to those from the Amazon and Cerrado regions, which were 49% and 11.2% respectively (Figure 2).

**Figure 3:**
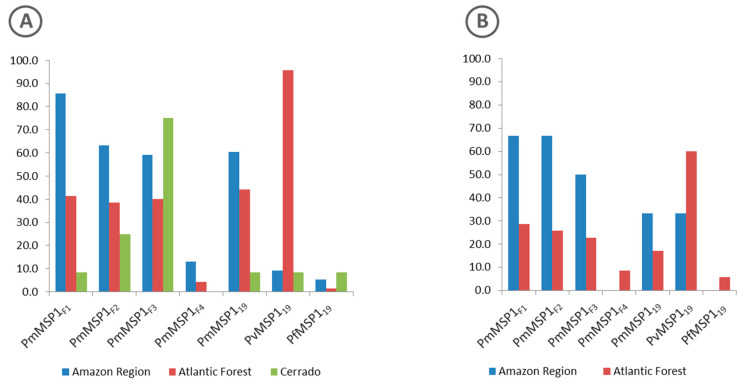
Percentage of positive sera from free-living (**A**) and captive (**B**) non-human primates of the Amazon Region (blue), Atlantic Forest (red) and Cerrado (green) regions to each of the MSP1 recombinant proteins.

Proteins with the highest rates of reactivity to the sera of free-living animals from all regions (Amazon, Atlantic Forest and Cerrado) were PmMSP1_F1_, PvMSP1_19_ and PmMSP1_F3_, respectively (Figure 3A).

As for the sera of animals in captivity, the percentage of sera samples that were reactive to any of the recombinant antigens that were tested was similar for those from the Atlantic Forest (35%) and from the Amazon region (31.6%) (Figure 2). The highest rates of reactive sera from such animals from the Amazon region were to proteins PmMSP1_F1_ and PmMSP1_F2_, while animals from the Atlantic Forest were most reactive to PvMSP1_19_ (Figure 3). Two sera of captive animals classified here as Atlantic Forest were positive to PfMSP1_19_ (Figure 3). However, these sera were from animals brought from the Amazon region and kept at the São Paulo Zoo.

Of the 164 positive samples for any of the *P. malariae* recombinant proteins, 34 (20%) reacted concomitantly to four proteins (PmMSP1_F1_, PmMSP1_F2_, PmMSP1_F3_ and PmMSP1_19_), while 61 (37%) sera reacted to only one of the proteins, with the lowest positivity to PmMSP1_F4_ (Supplementary information, Figure S2). 

### 3.3. Malaria Exposure History

**Figure 4:**
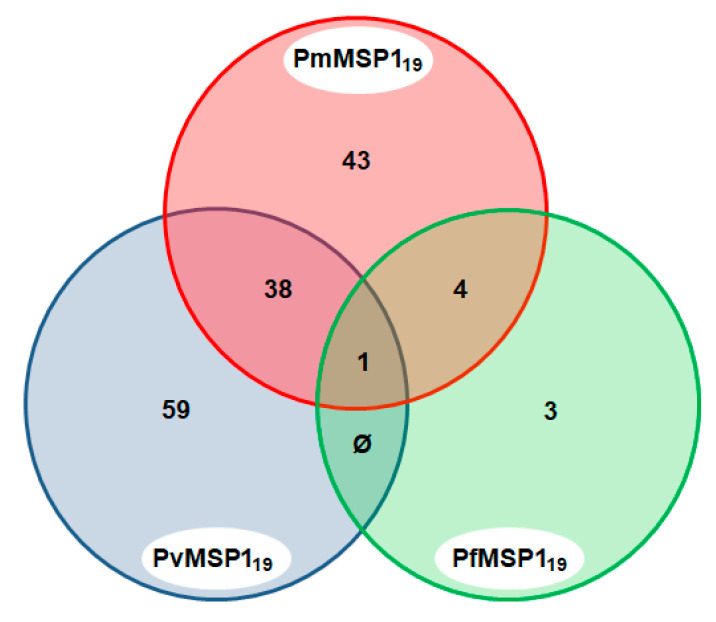
Venn diagram of sera that were positive to multiple *Plasmodium* species MSP1. Only sera that were reactive to the MSP1 C-terminal region of the three *Plasmodium* species, PmMSP1_19_, PvMSP1_19_ and PfMSP1_19_, are shown.

A total of 148 sera (29.9%) were found to be positive to the MSP1 C-terminal region of the different malaria parasites (PmMSP1_19_, PvMSP1_19_ and PfMSP1_19_). Of these, 43 (29.1%) were positive to more than one *Plasmodium* species: *P. falciparum* and *P. malariae* (2.7%); *P. vivax* and *P. malariae* (25.7%); and *P. falciparum, P. malariae* and *P. vivax* (0.7%), yielding evidence of the lifetime exposure of the animals to these *Plasmodium* species (Figure 4).

**Figure 5:**
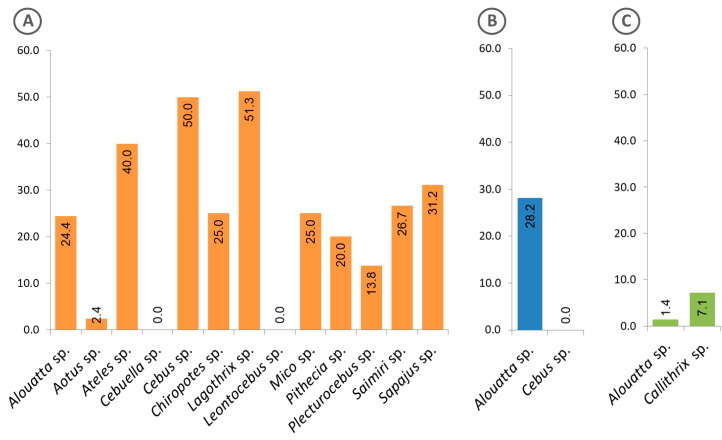
Frequency of free-living animals classified at genus level, with sera that were reactive to the *Plasmodium* recombinant MSP1. (**A**) Animals from the Amazon region, (**B**) Atlantic Forest and (**C**) Cerrado. The calculated percentages of positive sera are shown inside the bars.

Figure 5 shows the frequency of free-living animals with positive sera, classified by genus and region. Of the 13 genera of animals from the Amazon region, 11 presented positive sera (Figure 5A), while in the Atlantic Forest, only specimens of the genus *Alouatta* showed such positivity (Figure 5B). Interestingly, in the Cerrado region, sera from animals of another genus (*Callithrix*) were found positive, in addition to those of the genus *Alouatta* (Figure 5C). 

**Figure 6:**
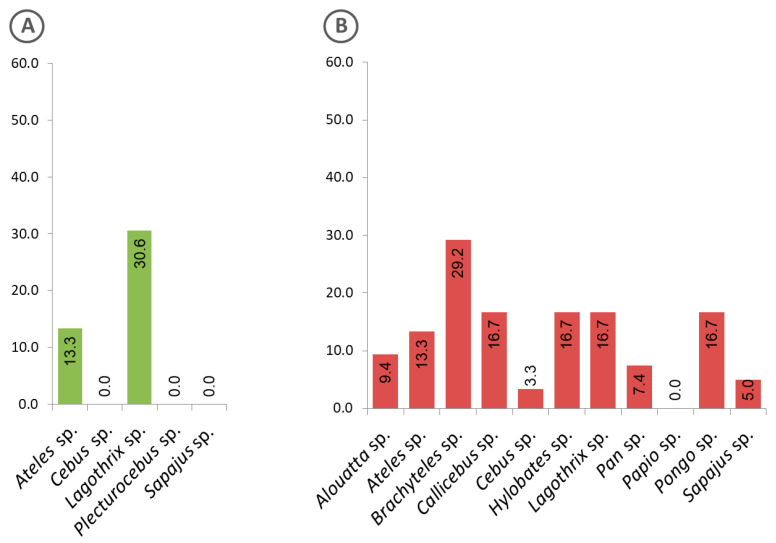
Frequency of captive animals with sera that were positive to the *Plasmodium* recombinant MSP1, classified at genus level. (**A**) Animals from the Amazon region, (**B**) Atlantic Forest.

Evidence of exposure to malaria parasites by animals kept in captivity at genus level is shown in Figure 6. While animals of the genus *Lagothrix* from the Amazon region showed the highest positivity rate (Figure 6A), those in the Atlantic Forest were of the genus *Brachyteles* (Figure 6B).

## 4. Discussion

Brazil is home to far more non-human primates than any other country; its 110 species account for about 27%, or one in every four, NHPs in the world. The Amazon region and the Atlantic Forest each house 20% of these taxa, including some endangered species [12,13].

NHPs have been shown to be infected by parasites from the genus *Plasmodium*, the etiological agent of malaria in humans, creating potential risks of zoonotic transmission and consequent public health concerns. Two species of simian malaria have been described in Brazil: *P*. *brasilianum*, a quartan malaria parasite genetically and morphologically similar to *P. malariae*, first described in bald uakari (*Cacajao calvus*) in the upper Amazon (Northern Brazil) [14] and *P*. *simium*, a tertian malaria parasite genetically and morphologically similar to *P. vivax*, described in a howler monkey (*Alouatta fusca*) in the state of São Paulo (southern Brazil) [15].

Recently, many studies have been performed to evaluate the presence of malaria parasites in NHPs, both in the Atlantic Forest and Amazon regions, aiming to understand their roles as reservoirs of malaria [4,7,8,16,17,18,19,20,21,22,23,24]. Some serological studies have also been carried out with the same aim but without using any *P. malariae* erythrocytic stage antigens [9,10,25]. In the present study, we used five recombinant antigens derived from the MSP1 protein of this parasite species. The recombinant proteins were designed as GST-fusion proteins and successfully used with human sera in a previous epidemiological study [6]. With these five recombinant proteins of *P. malariae* MSP1 (PmMSP1_F1,_ PmMSP1_F2,_ PmMSP1_F3,_ PmMSP1_F4,_ PmMSP1_19_), as well as with those of *P. falciparum* (PfMSP1_19_) and *P. vivax* (PvMSP1_19_), we found that about 40% of the NHP sera, from different areas in Brazil, were reactive to the recombinant antigens, with PmMSP1_F1_ being the most frequently detected. This might reflect the potentially high immunogenicity of this region of *P. malariae* MSP1, like those of *P. vivax* and *P. falciparum* MSP1, as described in humans [26,27]. Reactivity to the C-terminal portion of PmMSP1 (PmMSP1_19_) was also high, as expected from studies on the identical regions of *P. vivax* and *P. falciparum* MSP1 in humans [28]. Sera with antibodies to other regions of *P. malariae* MSP1 were also found, with the lowest frequency to PmMSP1_F4_, which might be due to its low binding capacity to the beads, as observed in the coupling efficiency assay.

Serological estimates correlate well with parasitological and entomological measurements in assessing transmission intensity and spatial and demographic risks to malaria [29,30]. The number of positive sera of animals from the Atlantic Forest was higher than that in the Amazon region, showing a likely higher *Plasmodium* circulation among NHPs from this area. This might also reflect the proximity of NHPs to humans at a higher populational density in the Atlantic Forest. The number of positive NHP sera from Cerrado was low, as expected, due to the low level of malaria incidence in Central Brazil. Another expected finding was the higher prevalence of anti- *P. vivax* and *P. malariae* antibodies in the sera of NHPs from the Atlantic Forest, as it has been demonstrated that *P. simium*, the equivalent of *P. vivax*, and *P. brasilianum*, the equivalent of *P. malariae,* are prevalent in this area, with the former being the most prevalent [18,19,31]. 

It is important to note here the higher diversity of NHPs from the Amazon region that was found to carry the anti-MSP1 antibodies, as compared to the diversity of NHPs from the Atlantic Forest, where only animals of the genus *Alouatta* have been found with such antibodies. This singularity has been observed since the description of the first *Plasmodium* infected animal [9,15,18,32,33]. These results might reflect the different species of mosquito vector present in the animals’ environments in each region, which in turn might reflect the different ecological niches of each component of the malaria system, the humans, the NHPs, the mosquito vectors and the parasites. More research is necessary for assessing all these relationships.

The seropositive NHPs indicate previous infections as well as the potential presence of infected animals in the surveyed areas, which can play a role in the maintenance of human malarias, making it more difficult to eliminate or even control the disease. Moreover, the illegal wildlife trade, the allocation of species for captivity or the effort to conserve them (translocation or reintroduction), promotes the movement of animals and may favor the transmission of disease [34,35]. Thus, it is necessary to carry out a rapid, inexpensive and effective test for *Plasmodium* diagnosis in NHPs in order to contribute to One Health surveillance. We have demonstrated that the MSP1 recombinant proteins used in this study are useful and important candidates to be included in diagnostic tools for the surveillance, and, ultimately, for the control or elimination, of malaria.

## 5. Conclusions

This study validates the use of recombinant proteins in multiplex immunoassays to detect IgG antibodies against the MSP1 protein of malaria parasites and demonstrates that this technique is an important tool for making serum-epidemiological surveys of malaria. Lastly, the high prevalence of NHPs with antibodies against *P. vivax* and *P. malariae* (as well as *P. falciparum* in the Amazon region) supports the hypothesis that these animals are potential reservoirs of malaria parasites and that NHPs must be considered in any measure for the control or elimination of the disease.

## Figures and Tables

**Table 1:** Average cut-off values obtained in the multiplex bead assay of *Plasmodium* recombinant proteins and non-human primate sera.

Malaria Species	Target Antigen	Signal Cut-Off Values (MFI) *
*P. malariae*	PmMSP1_F1_	252.6
*P. malariae*	PmMSP1_F2_	625.5
*P. malariae*	PmMSP1_F3_	465.0
*P. malariae*	PmMSP1_F4_	351.5
*P. malariae*	PmMSP1_19_	473.4
*P. vivax*	PvMSP1_19_	424.2
*P. falciparum*	PfMSP1_19_	471.7

* MFI, median fluorescent intensity. Average cut-off values obtained considering cut-off values of all the plates.

**Table 2:** Percentage of reactive sera (free-living and captive animals, *n* = 495) to the MSP1 recombinant proteins.

Recombinant Protein	Positive (%)	
PmMSP1_F1_	109	(22.0)
PmMSP1_F2_	91	(18.4)
PmMSP1_F3_	93	(18.8)
PmMSP1_F4_	16	(3.2)
PmMSP1_19_	86	(17.4)
PvMSP1_19_	98	(19.8)
PfMSP1_19_	8	(1.6)

## Supplementary Materials

The following are available online at https://www.mdpi.com/2076-0817/9/7/525/s1, **Figure S1.** Coupling efficiency of recombinant proteins to magnetic beads. Coupling efficiency of recombinant proteins to magnetic beads was assessed by using biotinylated rabbit anti-GST polyclonal IgG antibody (Abcam, Cambridge, MA, USA), followed by incubation with R-phycoerythrin-labelled streptavidin and fluorescence measurement with the BioPlex 200 instrument (Bio-Rad, Hercules, CA, USA), as described in Materials and Methods. **Figure S2.** Intersection chart of positive samples against *Plasmodium malariae* antigens. **Table S1**. Absolute and relative frequencies of positive free-living non-human primates by Bioplex assay per MSP1 protein. **Table S2**. Absolute and relative frequencies of positive captive non-human primates by Bioplex assay per MSP1 protein.
