# Supplementary material for: Naturally Acquired Humoral Immunity against Malaria Parasites in Non-Human Primates from the Brazilian Amazon, Cerrado and Atlantic Forest
Source: Pathogens. 2020 Jun 29;9(7):525. doi: 10.3390/pathogens9070525 (PMC7399928; doi:10.3390/pathogens9070525)
Supplement: Supplementary file 1 [file pathogens-09-00525-s001.pdf]

Supplementary Information

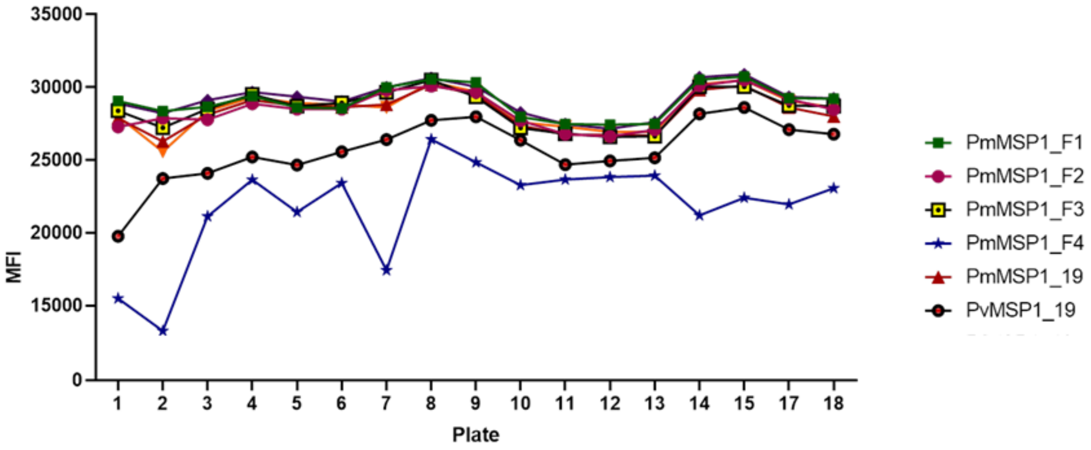

Figure S1. Coupling efficiency of recombinant proteins to magnetic beads. Coupling efficiency of recombinant proteins to magnetic beads was assessed by using biotinylated rabbit anti-GST polyclonal IgG antibody (Abcam, Cambridge, MA, USA), followed by incubation with R-phycoerythrin-labelled streptavidin and fluorescence measurement with the BioPlex 200 instrument (Bio-Rad, Hercules, CA, USA), as described in Materials and Methods.

Table S1. Absolute and relative frequencies of positive free-living non-human primates by Bioplex assay per MSP1 protein.

**Family**	**Species**	**Proteins**													
		**PmMSP1$_{F1}$**		**PmMSP1$_{F2}$**		**PmMSP1$_{F3}$**		**PmMSP1$_{F4}$**		**PmMSP1$_{19}$**		**PvMSP1$_{19}$**		**PfMSP1$_{19}$**	
**Aotidae**	***Aotus azarae***	**-**	**(0/10)**	**-**	**(0/10)**	**10.0**	**(1/10)**	-	(0/10)	10.0	(1/10)	-	(0/10)	-	(0/10)
	Aotus sp.	-	(0/4)	-	(0/4)	-	(0/4)	-	(0/4)	-	(0/4)	-	(0/4)	-	(0/4)
Atelidae	*Alouatta belzebul*	100.0	(1/1)	-	(0/1)	100.0	(1/1)	-	(0/1)	-	(0/1)	-	(0/1)	-	(0/1)
	Alouatta caraya	1.2	(1/86)	2.3	(2/86)	1.2	(1/86)	-	(0/86)	1.2	(1/86)	1.2	(1/86)	1.2	(1/86)
	Alouatta guariba	29.8	(17/57)	36.8	(21/57)	28.1	(16/57)	3.5	(2/57)	33.3	(19/57)	73.7	(42/57)	1.8	(1/57)
	Alouatta puruensis	35.7	(5/14)	42.9	(6/14)	28.6	(4/14)	-	(0/14)	28.6	(4/14)	7.1	(1/14)	-	(0/14)
	Alouatta sp.	22.6	(12/53)	11.3	(6/53)	22.6	(12/53)	1.9	(1/53)	22.6	(12/53)	47.2	(25/53)	-	(0/53)
	Ateles chamek	73.3	(11/15)	53.3	(8/15)	46.7	(7/15)	6.7	(1/15)	53.3	(8/15)	6.7	(1/15)	-	(0/15)
	Lagothrix cana	69.2	(9/13)	69.2	(9/13)	69.2	(9/13)	15.4	(2/13)	69.2	(9/13)	7.7	(1/13)	7.7	(1/13)
Callitrichidae	*Callithrix penicillata*	-	(0/21)	4.8	(1/21)	38.1	(8/21)	-	(0/21)	-	(0/21)	-	(0/21)	-	(0/21)
	Cebuella pygmaea niveiventris	-	(0/1)	-	(0/1)	-	(0/1)	-	(0/1)	-	(0/1)	-	(0/1)	-	(0/1)
	Leontocebus labiatus	-	(0/1)	-	(0/1)	-	(0/1)	-	(0/1)	-	(0/1)	-	(0/1)	-	(0/1)
	Leontocebus weddelli	-	(0/5)	-	(0/5)	-	(0/5)	-	(0/5)	-	(0/5)	-	(0/5)	-	(0/5)
	Mico melanurus	50.0	(1/2)	50.0	(1/2)	50.0	(1/2)	-	(0/2)	-	(0/2)	-	(0/2)	-	(0/2)
Cebidae	*Cebus albifrons*	100.0	(1/1)	100.0	(1/1)	100.0	(1/1)	-	(0/1)	-	(0/1)	-	(0/1)	-	(0/1)
	Cebus sp.	-	(0/1)	-	(0/1)	-	(0/1)	-	(0/1)	-	(0/1)	-	(0/1)	-	(0/1)
	Saimiri boliviensis	50.0	(1/2)	-	(0/2)	-	(0/2)	-	(0/2)	-	(0/2)	-	(0/2)	-	(0/2)
	Saimiri sp.	100.0	(1/1)	-	(0/1)	-	(0/1)	-	(0/1)	-	(0/1)	-	(0/1)	-	(0/1)
	Saimiri ustus	41.7	(5/12)	41.7	(5/12)	41.7	(5/12)	16.7	(2/12)	25.0	(3/12)	8.3	(1/12)	8.3	(1/12)
	Sapajus apella	57.9	(11/19)	36.8	(7/19)	26.3	(5/19)	21.1	(4/19)	36.8	(7/19)	10.5	(2/19)	5.3	(1/19)
	Sapajus cay	-	(0/1)	100.0	(1/1)	-	(0/1)	-	(0/1)	-	(0/1)	-	(0/1)	-	(0/1)
	Sapajus macrocephalus	33.3	(1/3)	33.3	(1/3)	33.3	(1/3)	33.3	(1/3)	33.3	(1/3)	-	(0/3)	-	(0/3)
Pitheciidae	*Chiropotes albinasus*	50.0	(1/2)	50.0	(1/2)	50.0	(1/2)	-	(0/2)	-	(0/2)	-	(0/2)	-	(0/2)
	Pithecia mittermeieri	40.0	(10/25)	16.0	(4/25)	24.0	(6/25)	-	(0/25)	36.0	(9/25)	-	(0/25)	4.0	(1/25)
	Plecturocebus brunneus	21.4	(3/14)	14.3	(2/14)	14.3	(2/14)	-	(0/14)	21.4	(3/14)	-	(0/14)	-	(0/14)
	Plecturocebus dubius	66.7	(2/3)	33.3	(1/3)	33.3	(1/3)	-	(0/3)	-	(0/3)	-	(0/3)	-	(0/3)
	Plecturocebus sp.	33.3	(2/6)	16.7	(1/6)	-	(0/6)	-	(0/6)	16.7	(1/6)	16.7	(1/6)	-	(0/6)

Table S2. Absolute and relative frequencies of positive captive non-human primates by Bioplex assay per MSP1 protein.

**Family**	**Species**	**Proteins**													
		**PmMSP1$_{F1}$**		**PmMSP1$_{F2}$**		**PmMSP1$_{F3}$**		**PmMSP1$_{F4}$**		**PmMSP1$_{19}$**		**PvMSP1$_{19}$**		**PfMSP1$_{19}$**	
**Atelidae**	***Alouatta caraya***	**-**	**(0/4)**	**-**	**(0/4)**	**-**	**(0/4)**	-	(0/4)	-	(0/4)	25.0	(1/4)	-	(0/4)
	Alouatta guariba	10.8	(4/37)	10.8	(4/37)	8.1	(3/37)	-	(0/37)	2.7	(1/37)	27.0	(10/37)	-	(0/37)
	Alouatta sp.	-	(0/7)	14.3	(1/7)	-	(0/7)	-	(0/7)	-	(0/7)	42.9	(3/7)	-	(0/7)
	Ateles chamek	28.6	(2/7)	14.3	(1/7)	14.3	(1/7)	-	(0/7)	14.3	(1/7)	-	(0/7)	-	(0/7)
	Ateles marginatus	50.0	(1/2)	-	(0/2)	-	(0/2)	-	(0/2)	50.0	(1/2)	-	(0/2)	-	(0/2)
	Ateles paniscus	50.0	(2/4)	25.0	(1/4)	-	(0/4)	25.0	(1/4)	25.0	(1/4)	-	(0/4)	-	(0/4)
	Ateles sp.	-	(0/2)	-	(0/2)	-	(0/2)	-	(0/2)	-	(0/2)	-	(0/2)	-	(0/2)
	Brachyteles arachnoides	25.0	(1/4)	25.0	(1/4)	50.0	(2/4)	-	(0/4)	25.0	(1/4)	50.0	(2/4)	-	(0/4)
	Lagothrix cana	20.0	(1/5)	40.0	(2/5)	40.0	(2/5)	-	(0/5)	40.0	(2/5)	40.0	(2/5)	-	(0/5)
	Lagothrix lagothricha	50.0	(1/2)	50.0	(1/2)	-	(0/2)	-	(0/2)	-	(0/2)	-	(0/2)	-	(0/2)
	Lagothrix poeppigii	100.0	(1/1)	100.0	(1/1)	-	(0/1)	-	(0/1)	-	(0/1)	-	(0/1)	-	(0/1)
Cebidae	*Cebus kaapori*	-	(0/3)	-	(0/3)	-	(0/3)	33.3	(1/3)	-	(0/3)	-	(0/3)	-	(0/3)
	Cebus sp.	-	(0/4)	-	(0/4)	-	(0/4)	-	(0/4)	-	(0/4)	-	(0/4)	-	(0/4)
	Sapajus apella	-	(0/22)	-	(0/22)	9.1	(2/22)	4.5	(1/22)	4.5	(1/22)	-	(0/22)	9.1	(2/22)
	Sapajus flavius	-	(0/2)	-	(0/2)	-	(0/2)	-	(0/2)	-	(0/2)	-	(0/2)	-	(0/2)
	Sapajus xanthosternos	-	(0/1)	-	(0/1)	-	(0/1)	-	(0/1)	-	(0/1)	-	(0/1)	-	(0/1)
Cercopithecidae	*Papio cynocephalus*	-	(0/1)	-	(0/1)	-	(0/1)	-	(0/1)	-	(0/1)	-	(0/1)	-	(0/1)
Hominidae	*Pan troglodytes*	-	(0/9)	11.1	(1/9)	-	(0/9)	-	(0/9)	-	(0/9)	33.3	(3/9)	-	(0/9)
	Pongo pygmaeus	-	(0/1)	-	(0/1)	-	(0/1)	-	(0/1)	-	(0/1)	100.0	(1/1)	-	(0/1)
Hylobatidae	*Hylobates lar*	100.0	(1/1)	-	(0/1)	-	(0/1)	-	(0/1)	-	(0/1)	-	(0/1)	-	(0/1)
Pitheciidae	*Callicebus nigrifrons*	-	(0/2)	-	(0/2)	50.0	(1/2)	-	(0/2)	-	(0/2)	50.0	(1/2)	-	(0/2)
	Plecturocebus sp.	-	(0/1)	-	(0/1)	-	(0/1)	-	(0/1)	-	(0/1)	-	(0/1)	-	(0/1)

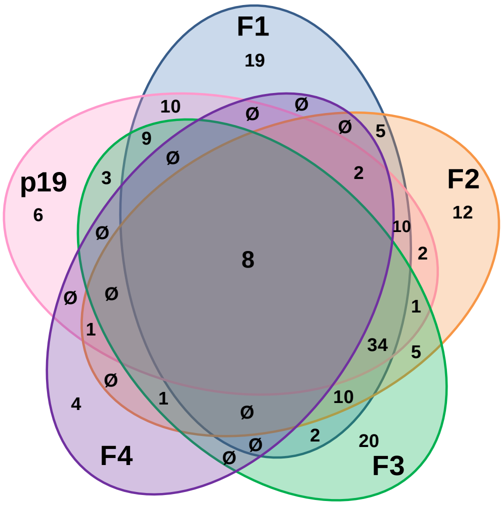

Figure S2. Intersection chart of positive samples against *Plasmodium malariae* antigens.
